# Efficient methane removal by thermophilic methanotrophs in a compost biofilter

**DOI:** 10.3389/fmicb.2026.1777601

**Published:** 2026-03-25

**Authors:** Geovanni Avila-Nuñez, Sergio Hernández-Jiménez, Patricia Ruiz-Ruiz, Sergio Revah

**Affiliations:** 1 Departamento de Procesos y Tecnología, Universidad Autónoma Metropolitana-Cuajimalpa, Cd. de México, Mexico; 2 Center for Microbial Ecology and Technology (CMET), Faculty of Bioscience Engineering, Ghent University, Gent, Belgium

**Keywords:** biofiltration, compost biofilter, greenhouse gases, methane oxidation, *Methylocaldum*, thermophilic methanotrophs

## Abstract

Methane (CH_4_) is a potent greenhouse gas, and biofiltration has proved to be a valuable strategy for treating diffuse emissions from composting and landfill systems. This study characterized the activity of a thermophilic methanotrophic consortium enriched from compost. It also evaluated the consortium’s performance, under controlled conditions, in a liquid stirred-tank bioreactor and a compost biofilter for 90 and 65 days, respectively. The consortium, specifically the thermophilic methanotroph *Methylocaldum*, showed robust CH_4_ oxidation at 50 °C, attained a maximum elimination capacity (EC) of 342 mg CH_4_/kg compost·h with 75% removal efficiency (RE), and maintained an EC of ~250 mg CH_4_/kg compost·h from day 28 with 100% RE. Heat treatment of compost followed by inoculation with the methanotrophic consortium enhanced start-up, reduced microbial competition, and improved stability. Oxygen availability and moisture were identified as critical parameters, while nutrient supplementation had minimal effect. Analysis of respiratory coefficients indicated that heterotrophic microorganisms competed for oxygen, influencing CH_4_ removal efficiency. Compared with mesophilic systems, the thermophilic biofilter achieved a higher EC, demonstrating the microbes’ ability to adapt to higher temperatures. These findings confirm the potential of thermophilic biofiltration for effective CH_4_ migration and provide guidance for optimizing design and operational strategies in large-scale applications.

## 1 Introduction

Methane (CH_4_) is a potent greenhouse gas, with a global warming potential of ~27.9 times greater than that of carbon dioxide (CO_2_) over a 100-year period, (GWP-100), and an atmospheric lifetime of ~12 years, making it a critical target for near-term climate mitigation (IPCC, 2023). Over 60% of global CH_4_ emissions are anthropogenic, primarily coming from agriculture, waste management, and energy production (Skeie et al., 2023). While the remaining emissions originate from natural sources, releasing ~218 Tg CH_4_ annually, the main sink is atmospheric chemical oxidation (~90%) (Saunois et al., 2020). Methanotrophs are a unique group of microorganisms that grow on CH_4_, acting as a natural sink like a filter. Biological oxidation represents ~5%, attenuating CH_4_ emission in various ecosystems (Knief, 2015), even at high temperatures, with methanotrophs inhabiting these environments (Tsubota et al., 2005; Houghton et al., 2023). Oxidation of CH_4_ by microbes plays a key role in mitigating CH_4_ emissions from both natural and anthropogenic sources.

Aerobic methanotrophs catalyze the oxidization of CH_4_, sequentially producing methanol, formaldehyde, formate, and ultimately CO_2_. For carbon assimilation, formaldehyde is incorporated through the ribulose monophosphate (RuMP) pathway or the serine cycle, while CO_2_ can be assimilated through the Calvin–Benson–Bassham (CBB) cycle (Semrau et al., 2018; Ahmadi and Lackner, 2024). The majority of described aerobic methanotrophs belong to the Alpha- and Gammaproteobacteria, with a few representative thermophilic lineages, while others have been identified within Verrucomicrobia in geothermal and volcanic environments (Smith and Wrighton, 2019; Ahmadi and Lackner, 2024). Despite their ecological and environmental significance, thermophilic methanotrophs remain insufficiently explored (Houghton et al., 2019), even though relevant man-made systems such as composting and landfill covers, sustain thermophilic CH_4_ oxidation (Eshinimaev et al., 2004; Jäckel et al., 2005).

Biofiltration has emerged as a promising technology to treat diffuse, low-concentration CH_4_ emissions. These systems employ porous packed beds, mostly compost-based beds, that support microbial biofilms where CH_4_ diffuses and is then oxidized. Numerous abiotic factors, such as temperature, moisture, nutrient availability, and packing material, strongly influence the performance of these systems (Nikiema et al., 2007; Ménard et al., 2012; La et al., 2018). While conventional biofilters typically operate optimally at 20–36 °C (Nikiema et al., 2007; Gómez-Borraz et al., 2017), the exothermic nature of CH_4_ oxidation can raise the internal temperature up to 50–60 °C (Gómez-Borraz et al., 2025). Higher temperature levels in biofilters have shown to induce accelerated bed drying and impaired activity (Morales et al., 2003; Huete et al., 2018).

Nonetheless, evidence indicates that CH_4_ oxidation can be sustained at higher temperatures, signifying the presence and potential utility of thermophilic methanotrophs (Scheutz et al., 2017; Fjelsted et al., 2020). In engineered man-made systems (Table 1), heat generation and elevated temperature levels have been reported in large-scale case studies. The increased temperature is due to the exothermic nature of organic waste decomposition, compost maturation, and CH_4_ oxidation (Jäckel et al., 2005; Coccia et al., 2013). Adapted methanotrophs are particularly advantageous under these conditions. Although the aqueous solubility of CH_4_ and oxygen (O_2_) decreases with increasing temperature, potentially limiting methanotrophic growth, thermotolerant and thermophilic methanotrophs possess metabolic and physiological adaptations that enable their survival and growth under elevated temperatures (Houghton et al., 2019; Delherbe et al., 2024). Among these, *Methylocaldum* spp. are considered thermophiles and are frequently identified as dominant methanotrophs at high temperatures (Reddy et al., 2019; Yang et al., 2021).

**Table 1:** Review of studies on reporting CH_4_ uptake at high temperatures.

Reference	System	CH_4_ concentration	Elimination capacity	Temperature range	Removal efficiency
Gómez-Borraz et al. (2025)	Compost biofilter	2–8%	75 g/m^3^·h	30–60 °C	75–100%
Yang et al. (2021)	Biocover	16–31%	–	20–49 °C	96–99%
Fjelsted et al. (2020)	Compost biofilter	5–10%	~38 g/m^3^·h	50–60 °C	~58%
Huete et al. (2018)	Compost biofilter	2–4%	~42 g/m^3^·h	33–51 °C	~70%
Scheutz et al. (2017)	Biocover	<14%	–	~55 °C	~81–100%
Jäckel et al. (2005)	Compost piles	<0.1–10%	–	53–78 °C	46–98%

The systematic characterization of the performance, stability, and operational limits of compost biofiltration at high temperatures remains limited. This gap exists despite studies on the thermophilic methanotrophs, as biological activity depends on biotic factors, such as competition from heterotrophs in the compost, and abiotic conditions, such as water content, gas and heat transfer, and gas flow distribution. This study addresses this gap by evaluating the activity of a thermophilic methanotrophic consortium (TMC) under controlled conditions in a lab-scale compost biofilter. Methane oxidation and respiration were assessed under varying operational parameters—including CH_4_ concentration, temperature, moisture, nutrient availability, and feeding regimes—to investigate the performance and resilience of thermophilic methanotrophs in engineered biofiltration systems.

## 2 Materials and methods

### 2.1 Enrichment of the thermophilic methanotrophic consortium

In this study, samples were taken from the bottom of a compost biofilter treating CH_4_ (Gómez-Borraz et al., 2025), which attained sustained temperatures above 50 °C. The TMC was enriched using triplicates of 2 g compost samples that were resuspended in 23 mL medium of nitrate mineral salts (NMS, Section 2.2) in 125 mL serological bottles and incubated at 50 °C and 150 rpm. Cultures were grown for 14 days and fed every 24 h with 15% CH_4_ v/v (99% v/v, Praxair, México) in air. From the first enrichment, triplicates of four serial 1:10 dilutions were prepared in 25 mL vials containing 5 mL of medium, and the same growth conditions were maintained. Growth was observed up to the third dilution, which was then pooled and used as inoculum for the TMC.

### 2.2 Growth conditions of thermophilic methanotrophic consortium

The NMS medium composed of (g/L): KNO_3_ (1.0), MgSO_4_·7H_2_O (0.2), CaCl_2_·2H_2_O (0.067), KH_2_PO_4_ (0.348), Na_2_HPO_4_·12H_2_O (0.242), FeSO_4_·7H_2_O (0.005), and a trace elements solution (0.1% v/v) containing CuSO_4_·5H_2_O (0.22), ZnSO_4_·7H_2_O (0.44), MnSO_4_·H_2_O (0.15), H_3_BO_3_ (0.1), CaCl_2_·2H_2_O (0.18), and Na_2_MoO_4_·2H_2_O (0.06). The pH of the medium was adjusted to 7.0. Maintenance cultures of the TMC in liquid were carried out in 125 mL serological bottles with butyl rubber stoppers, containing 25 mL of culture volume. The headspace was supplemented with 15% CH_4_ and incubated at 50 °C with shaking at 150 rpm.

### 2.3 Bioreactor culture

A 3 L stirred tank bioreactor (Applikon Biotechnology, The Netherlands) containing 1.3 L of NMS medium was inoculated with 0.2 L of TMC culture at 50 °C and agitated at 200 rpm. The reactor headspace was replenished every 3 h with a gas mixture containing 15% CH_4_ in air. Inlet and outlet gas concentrations (CO_2_, O_2_, and CH_4_) were periodically measured using gas chromatography with a thermal conductivity detector (GC–TCD). Liquid samples (40 mL) were collected to evaluate biomass and nitrate (NO_3_^−^) concentrations. The kinetics of CH_4_ consumption were monitored periodically, and the maximum specific CH_4_ consumption rate was estimated by fitting the CH_4_ concentration data, obtained through gas chromatography with a flame ionization detector (GC–FID), to the Gompertz model (Acuña et al., 1999). After the cultivation phase, 2 h activity assays were conducted in the reactor under varying conditions (Table 2), including nutrient supplementation (addition of fresh medium with 100 mg/L NO_3_^−^), increased agitation speed, and after detaching the biomass that had adhered to the wall, stirrer, and baffles of the bioreactor.

**Table 2:** Experiments for analyzing effects on activity of TMC in bioreactor culture.

Experiment	Added nutrients	Stirring (RPM)	Biomass
E1	No	200	Suspended
E2	No	500	Suspended
E3	Yes	200	Suspended
E4	Yes	500	Suspended
E5	Yes	200	Accumulated^*^
E6	Yes	500	Accumulated^*^

* Refers accumulated as biomass suspended and collected from wall, stirrer, and baffles of the bioreactor.

### 2.4 Compost biofilter media and CH_4_ kinetics

Compost derived from pruning waste was collected locally in Cuajimalpa, Mexico City, in October 2022. The compost was sieved to remove particles larger than 1 cm. Its physicochemical properties including moisture content, pH, apparent density, particle size distribution, water-holding capacity, and soluble carbon/nitrogen (C/N) ratio, were analyzed following standard procedures (Supplementary Table S1). Solid cultures were prepared in 500 mL bottles containing ~65 g (≈100 mL) of compost. Each bottle was sealed with a rubber-stoppered cap for gas sampling and incubated for 7 days. The headspace gas was refreshed every 24 h with a gaseous mixture of 15% CH_4_ in air. The cultures were aseptically inoculated with 10 mL of TMC biomass (0.2 g _DW_/L), corresponding to 30 mg _DW_ per kg of compost. The moisture levels were maintained by periodically weighing the bottles and adding NMS medium to compensate for weight loss.

Microbial activity was evaluated by measuring heterotrophic respiration (CO_2_ production and O_2_ consumption) and CH_4_ oxidation kinetics at the start of incubation and on Days 3 and 7 in duplicate. The following conditions were tested: (a) heat-treated verses untreated compost; (b) inoculated versus uninoculated compost; (c) incubation temperatures of 50 °C versus 60 °C; and (d) with or without CH_4_ supplementation. To reduce the activity of the indigenous methanotrophs, the compost was subjected to heat treatment at 95 °C for 30 min, followed by incubation at 50 °C for 24 h, and this cycle was further repeated for three consecutive days.

### 2.5 Description of the lab-scale biofilter

**Figure 1:**
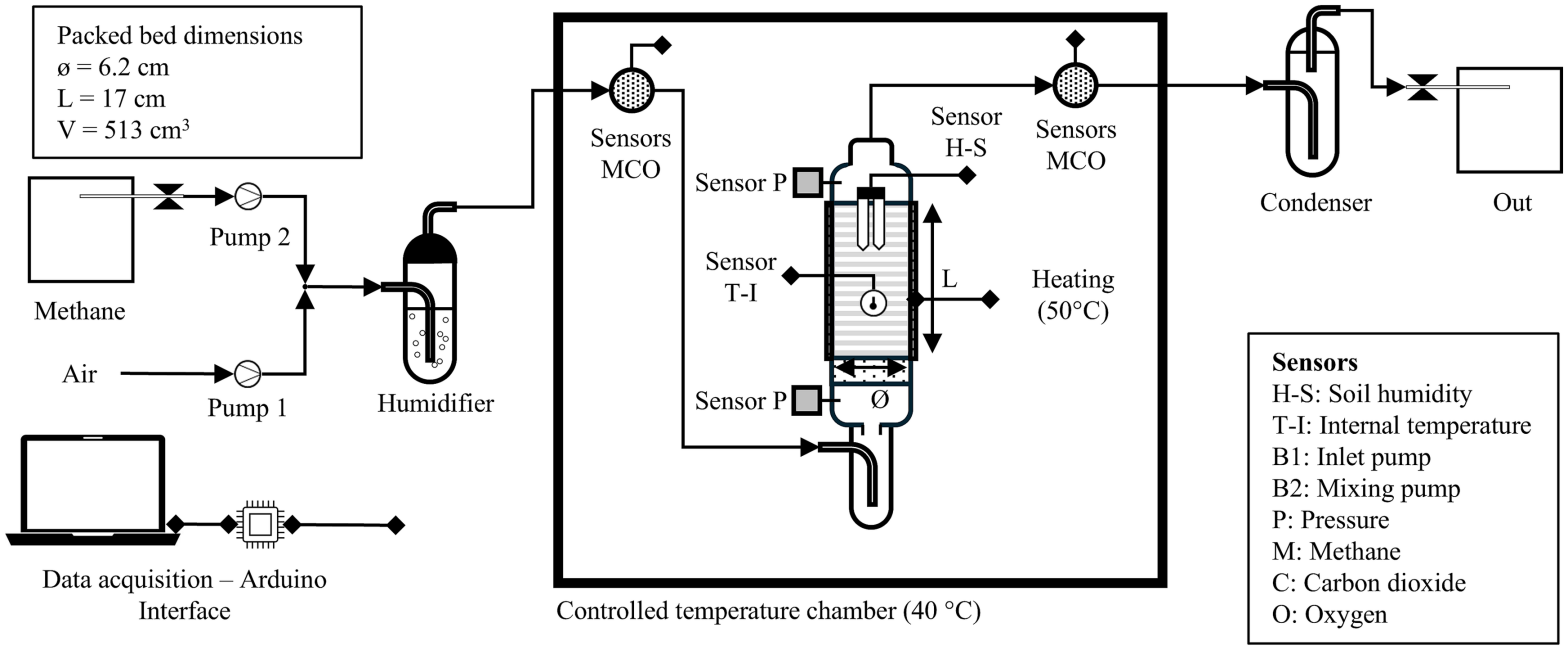
Experimental set-up of lab-scale biofilter.

The experimental biofiltration system, as shown in Figure 1, consisted of a cylindrical column with an effective packed with 0.5 L bed volume of compost. Set-up included a humidifier at the inlet and a condenser at the outlet. A container for leachate collection and gas supply was also placed at the bottom. The biofilter inoculum was acclimated with 15% CH_4_ in air, replaced daily in a solid culture with 100 mL compost in 500 mL bottles. The inoculum was maintained for 75 days to acclimate the TMC to compost medium at 50 °C and 60% humidity. Gas consumption and production of CH_4_ was monitored for evaluating methanotrophic activity and respiratory coefficient (Supplementary Figure S1). The biofilter was packed with a mixture of heat-treated fresh compost and the inoculated compost in a ratio of 1:5.

The biofilter gas inlet was supplied with two peristaltic pumps to mix air and CH_4_ to the desired concentrations. Gas concentration, pressure (MPS20N0040D-S), temperature (DS18B20), and packed bed humidity (SEN-HS-CAP) were monitored in real time with sensors connected to Arduino IDE and data acquisition was performed in Data Streamer Excel. All sensors were calibrated under operational conditions in a controlled temperature chamber (40 °C). The biofilter was maintained at 50 °C using a silicon belt heater. Inlet and outlet gas concentrations were monitored with specific sensors for CO_2_ (MG811), O_2_ (SEN0322), and CH_4_ (MQ4). Technical specifications of all sensors used in this study are summarized in Supplementary Table S2.

Four inlet CH_4_ operating conditions were assayed by varying the CH_4_ concentration and the empty bed residence time (EBRT) and consequently, the inlet load (IL). During the first condition (days 0–13), the inlet CH_4_ concentration was 5% with a residence time of 31 min (~90 mg CH_4_/kg compost·h at 16 mL/min). In the second condition (days 14–27), the inlet CH_4_ concentration was increased to 10% (~140 mg CH_4_/kg compost·h at 16 mL/min). The third condition (days 28–41) was carried out with 10% CH_4_ and a residence time of 18 min (~250 mg CH_4_/kg compost·h at 28 mL/min). The fourth condition (days 42 65), was operated with a residence time of 9 min (~450 mg CH_4_/kg compost·h at 55 mL/min).

### 2.6 Thermophilic conditions experiments

Experiments were performed using active compost from the biofilter. The methanotrophic and heterotrophic (respiration) activity were analyzed to determine the net effect of abiotic factors on the performance of thermophilic biofiltration. All cultures were incubated (in duplicate) at 50 °C and supplied with fresh air and 10% CH_4_ (methanotrophy experiments) in the headspace. To test the effect of moisture (40 and 60%), a 100 mL active compost sample was supplemented with sterile distilled water, made up to the desired water content, and incubated in 500 mL rubber stoppered bottles. The effect of added nutrients was evaluated in 125 mL serological rubber stoppered bottles containing 25 mL of culture and centrifuged at 150 rpm. The compost was homogenized in sterile distilled water to obtain a suspension at a concentration of 150 g _DW_ compost/L. A concentrated solution of NO_3_^−^, SO_4_^2−^, PO_4_^3−^, and trace metals (Cu^2+^, Mg^2+^, Fe^3+^, Ca^2+^, and others) was added to reach the final concentrations of the NMS medium (see above). The treatments were compared with controls without nutrient and with the original compost. For testing the effect of carbohydrates, the TMC was incubated in liquid medium as described above, with glucose or fructose concentrations of 10 mg/L, and a control without carbohydrates.

### 2.7 Microbial composition analysis by 16S rRNA gene sequencing

The bacterial diversity of the TMC enrichment was evaluated by DNA extraction and Nanopore sequencing of the16S rRNA gene (Secoya Labs, México). The DNA extraction was performed with a CH_4_ growing TMC culture used in inoculated bioreactor and biofilter experiments, and the biomass was concentrated by centrifugation. A biomass pellet of approximately 250 μL was processed according to the DNeasy PowerSoil DNA Isolation Kit protocol (Qiagen Sciences). For PCR amplification of 16S rRNA gene, universal primers Bac8F (5′-AGAGTTTGATCCTGGCTCAG-3′) and 1492R (5′-GGTTACCTTGTTACGACTT-3′) were used (Weisburg et al., 1991). Raw sequences were preprocessed and filtered through Qiime2 v2025.4 (Boylen et al., 2019), resulting in reads with a mean read length of 1223 pb. The VSEARCH plug-in was used to cluster sequences with standard parameters (Rognes et al., 2016). The Operational Taxonomic Units (OTUs) generated were subjected to taxonomic annotation (percent identity 0.9) using BLAST+ (Camacho et al., 2009) with a curated reference database of 16S rRNA sequences SILVA database (Quast et al., 2013).

### 2.8 Analytical procedures

Dry weight (DW) was determined by vacuum filtering the liquid culture samples through a pre-weighted 0.2 μm pore size cellulose acetate membrane (Sartorius), followed by drying at 60 °C for 24 h. The liquid filtrate was recovered and preserved at 4 °C. The nitrogen concentration (NO_3_^−^) in the filtrate was measured by UV-spectrophotometry at 220 nm after diluting 2 mL of sample at a 1:100 ratio and acidifying with 1 mL of 1 M HCl.

The CH_4_ concentration in bioreactor culture was analyzed using an Agilent 7890B gas chromatograph equipped with an HP-5 column (Agilent 19091J-413E) and a flame ionization detector (FID). Nitrogen was used as carrier gas at a rate of 2.0 mL/min and the temperatures of the injector, oven, and detector were maintained at 100 °C, 80 °C, and 200 °C, respectively. Gas composition (CO_2_, O_2_, and CH_4_) was analyzed using 250 μL gas samples. The analysis was conducted in a Gow-Mac Series 580 gas chromatograph with a CTR1 column (Alltech, USA) equipped with a thermal conductivity detector (TCD). Helium was used as carrier gas at 70 mL/min and the temperature were set at 50 °C in the injector, 40 °C in the column, and 115 °C in the detector.

Analysis of the compost properties were conducted in accordance with the Mexican official standard NMX-FF-109-SCFI-2008, with minor modifications. Moisture was determined gravimetrically using pre-weighted aluminum trays. Compost samples (10 g) were weighed and dried at 105 °C for 24 h; the difference between the initial and final weight was used for calculating the moisture content. The pH of the compost was measured with an SG200C electrode (Sensorex, USA) using an aqueous suspension of compost and distilled water (1:5, w/v). Apparent density was determined using a 100 mL graduated cylinder after drying at 105 °C for 24 h, while packed density was measured after gentle vertical tapping. Both were expressed as weight per unit volume. Water-holding capacity was determined by gravimetry based on the amount of water retained at equilibrium and at saturation, calculated as the difference between the initial wet weight and the final dry weight after drying at 105 °C for 24 h. Particle size distribution was determined by dry sieving. Triplicate samples were weighed initially, and the fractions retained on sieves with mesh sizes of 4, 8, 10, 20, 30, 50, and 100 (U.S. Standard Sieve Series) were used to calculate the particle size distribution (Supplementary Figure S2).

Soluble C/N was determined using the saturated extract method and expressed as the ratio of total organic C to total N in water. The saturated extract was obtained by saturating compost with distilled water and vacuum-filtering the mixture through cellulose filter paper (Whatman, Grade 1). Total carbon (TC) and inorganic carbon (IC) were measured with a TOC-L CSH analyzer (Shimadzu, Japan) equipped with a non-dispersive infrared (NDIR) detection system, according to the oxidative catalytic combustion method. Acidification was performed with 0.1 M HCl, and the combustion temperature was set at 680 °C. Total organic carbon (TOC) was calculated as the difference between TC and IC. Total nitrogen (TN) was determined with the TNM-L chemiluminescence module of the same instrument.

### 2.9 Statistical analyses and calculations

The performance of biofiltration was evaluated using elimination capacity (EC) and removal efficiency (RE). In addition, inlet load (IL) and EBRT were used to characterize the operating regimes. Calculations were performed as described in Table 3.

**Table 3:** Calculation of parameters for characterizing and evaluating the biofiltration system.

Parameter	Units	Equation
Elimination capacity (EC)	mg CH_4_/kg compost·h	$\mathrm{EC}=\frac{(C_{\text{inlet},\mathrm{mg}/L\;{\mathrm{CH}}_{4}}-C_{\text{outlet},\mathrm{mg}/L\;{\mathrm{CH}}_{4}})\cdot Q_{\text{inlet}}}{V_{\mathrm{bed}}\cdot \rho_{\text{packed}\;\mathrm{bed}}}$
Removal efficiency (RE)		$\mathrm{RE}=\frac{\left(C_{\text{inlet},\mathrm{mg}\;{\mathrm{CH}}_{4}}-C_{\text{outlet},\mathrm{mg}\;{\mathrm{CH}}_{4}}\right)}{C_{\text{inlet},\mathrm{mg}\;{\mathrm{CH}}_{4}}}$
Inlet load (IL)	mg CH_4_/kg compost·h	$\mathrm{IL}=\frac{C_{\text{inlet},\mathrm{mg}/L\;{\mathrm{CH}}_{4}}\cdot Q_{\text{inlet}}}{V_{\mathrm{bed}}\cdot \rho_{\text{packed}\;\mathrm{bed}}}$
Empty-bed residence time (EBRT)	min	$\text{EBRT}=\frac{V_{\mathrm{bed}}}{Q_{\text{inlet}}}$

Where C_inlet_ and C_outlet_ are the CH_4_ concentrations (mg/L) at the biofilter inlet and outlet, respectively; Q_inlet_ is the inlet flow rate of CH_4_ (L/h); V_bed_ is the packed bed volume (L), and p_acked bed_ is the packed bed density (kg/L) of the compost.

The results are presented as the average ± standard deviation. An ANOVA with LSD post-hoc analysis was performed to assess the statistically significant differences between the experiments. A significance level of 0.05 was used to calculate the *p*-value.

## 3 Results and discussion

### 3.1 Growth dynamics of the thermophilic methanotrophic consortium

**Figure 2:**
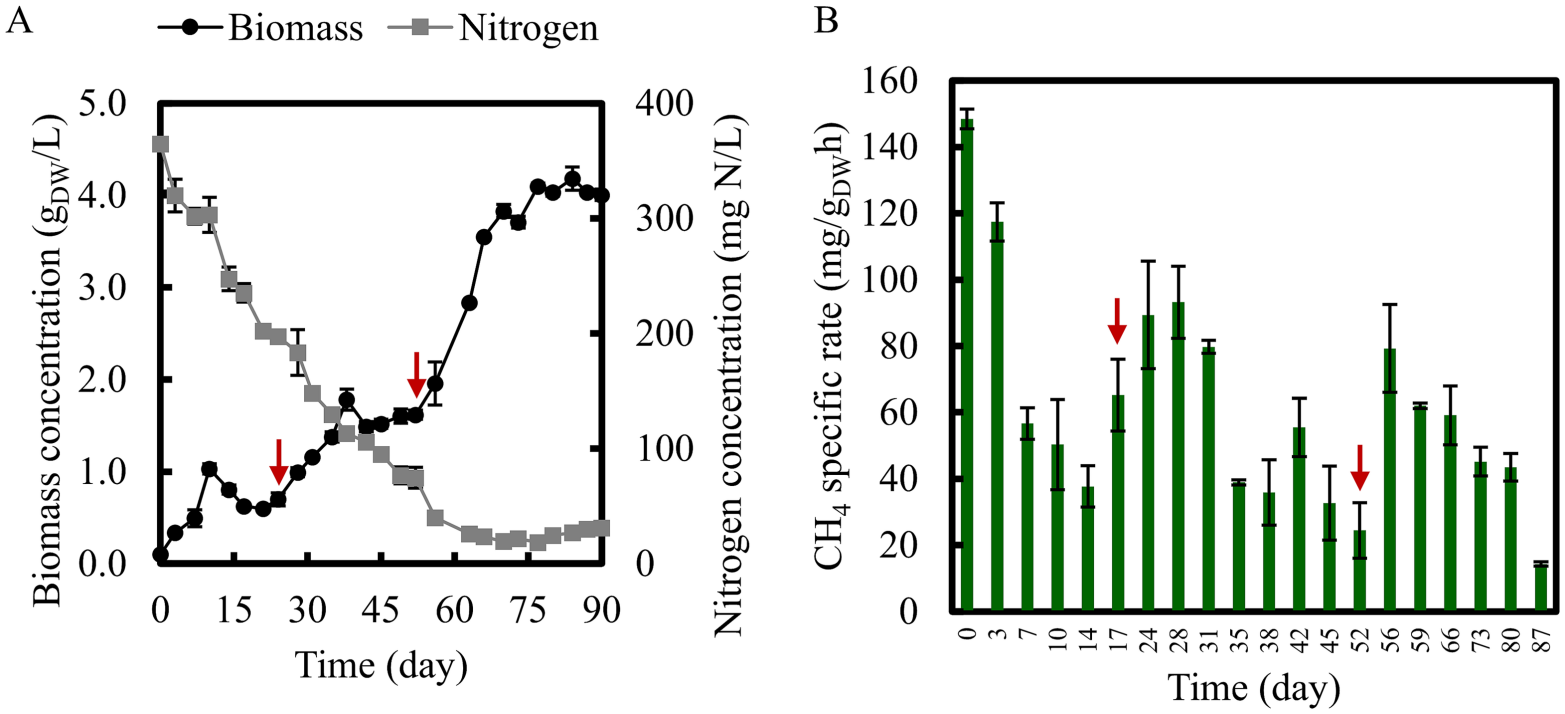
Bioreactor culture **(A)** biomass and nitrogen concentration and **(B)** CH_4_ specific consumption rate determined by closed headspace kinetics. Arrows indicate the time points of nutrient supplementation.

The TMC culture was cultivated in a bioreactor with an initial biomass concentration of 0.1 g _DW_/L (Figure 2A). During the first 10 days, biomass concentration increased to 1.0 ± 0.06 g _DW_/L and subsequently declined to approximately 0.7 g _DW_/L, despite nitrogen still being available and the pH maintained at 6.8. Biomass growth was re-established on Day 24 following the addition of fresh nitrogen-free NMS medium, reaching 1.7 ± 0.11 g _DW_/L. A similar growth decline was observed on Day 38, which was reversed after the addition of the nitrogen-free medium. Concurrently, CH_4_ specific consumption rates increased following each medium addition (Figure 2B). As observed in bioreactor culture of the TMC, repeated biomass decline and recovery following medium addition suggest that nutrients other than the nitrogen source may have limited microbial growth and activity. Trace metals such as copper may have played a critical role in community metabolism, as copper is essential for the CH_4_ oxidation in methanotrophs due to its involvement in particulate methane monooxygenase activity (Semrau et al., 2018). Despite the availability of nitrogen, the depletion of such micronutrients may explain the recurrent reduction in biomass and microbial activity observed during prolonged operation.

By Day 70, corresponding to the onset of the stationary phase, suspended biomass reached 3.8 ± 0.07 g _DW_/L, after which further growth was constrained by nitrogen availability. On Day 90, suspended biomass increased slightly to 4.0 ± 0.06 g _DW_/L, while total biomass reached 7.3 ± 0.23 g _DW_/L upon inclusion of biofilm attached to the bioreactor wall, baffles, and impeller. During the stationary phase, yields shifted markedly, with increased CO_2_ production and O_2_ consumption. During the growth phase (Supplementary Figure S3), yields were 0.30 mol CO_2_/mol CH_4_ and 0.62 mol O_2_/mol CH_4_, whereas during the stationary phase, the yield increased to 0.47 mol CO_2_/mol CH_4_ and 1.17 mol O_2_/mol CH_4_, doubling the O_2_ consumption per CH_4_ consumed. This variation can be attributed to a metabolic shift from growth to maintenance in the stationary phase.

**Figure 3:**
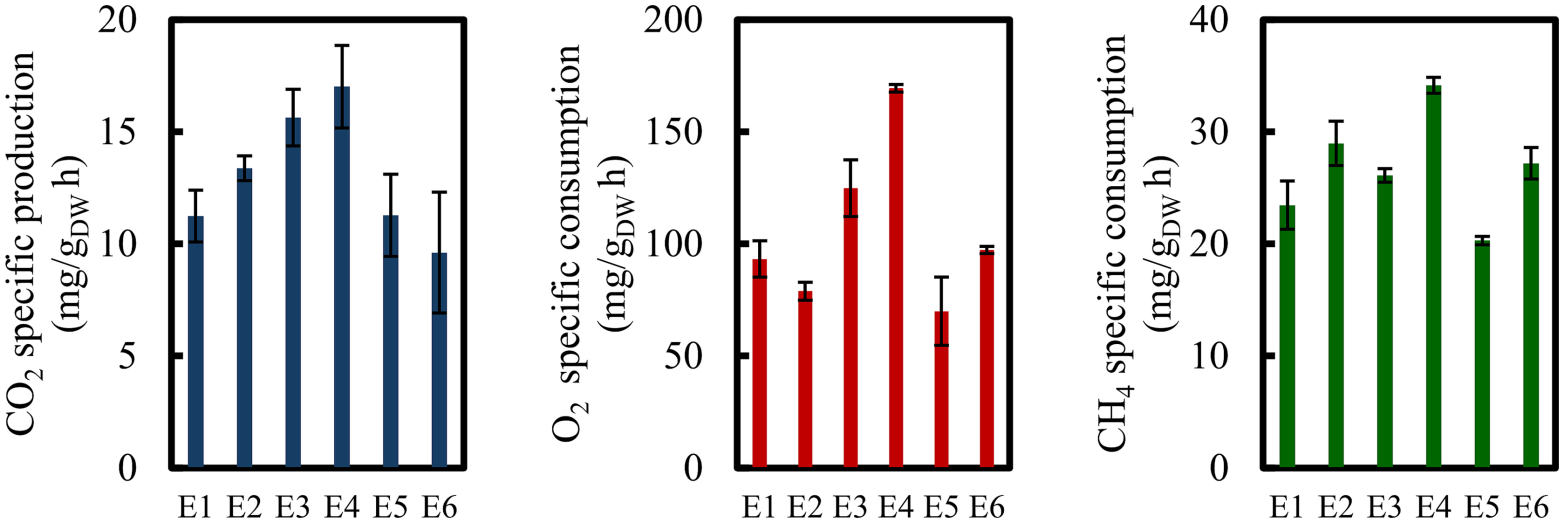
Maximum specific CH_4_ consumption and production rates of CO_2_, O_2_, and CH_4_, in experiments for evaluating the activity of TMC in bioreactor culture. E1 to E6 conditions are depicted in Table 2.

To evaluate the effects of nutrient availability, stirring, and biomass accumulation on metabolic activity, the experiments depicted in Table 2 were conducted. The results shown in Figure 3 indicated an improvement in specific activity upon nutrient addition and increased agitation with the highest values observed when only suspended biomass was in medium bioreactor. Under these conditions (E4), specific activities reached 17 ± 1.8, 169 ± 1.6, and 34 ± 0.7 mg/g _DW_ h for CO_2_, O_2,_ and CH_4_, respectively. Although total biomass in the medium was higher after biofilm detached and resuspension, no corresponding improvement in specific activity was observed. In addition, biofilm formation was evident within the bioreactor and likely contributed to the apparent decrease in suspended biomass observed after Days 10 and 38. The attachment of biomass to reactor walls, baffles, and impeller effectively reduced the suspended biomass, specifically metabolically active cells, as reflected by the discrepancy between the suspended and total biomass. Moreover, biofilm formation may have negatively affected activity due to increased diffusion limitations for substrates and nutrients (Stewart, 2003), particularly for the sparingly soluble gases such as CH_4_ and O_2_.

Community analysis of the TMC (Supplementary Figure S4) revealed that the genus *Methylocaldum* dominated the microbial community. Members of this genus are widely distributed across diverse environments, including several thermophilic and thermotolerant species (Trotsenko et al., 2009; Houghton et al., 2019), and are likely the primary drivers of CH_4_ oxidation at high temperatures. The presence of *Steroidobacter* as the second most abundant genus suggests potential metabolic interactions within the consortium, as this chemotrophic bacterium is capable of aerobic growth on a variety of complex carbon substrates, such as biofilm-derived exopolysaccharides (EPS) (Sakai et al., 2014).

### 3.2 CH_4_ oxidation in compost solid cultures

**Figure 4:**
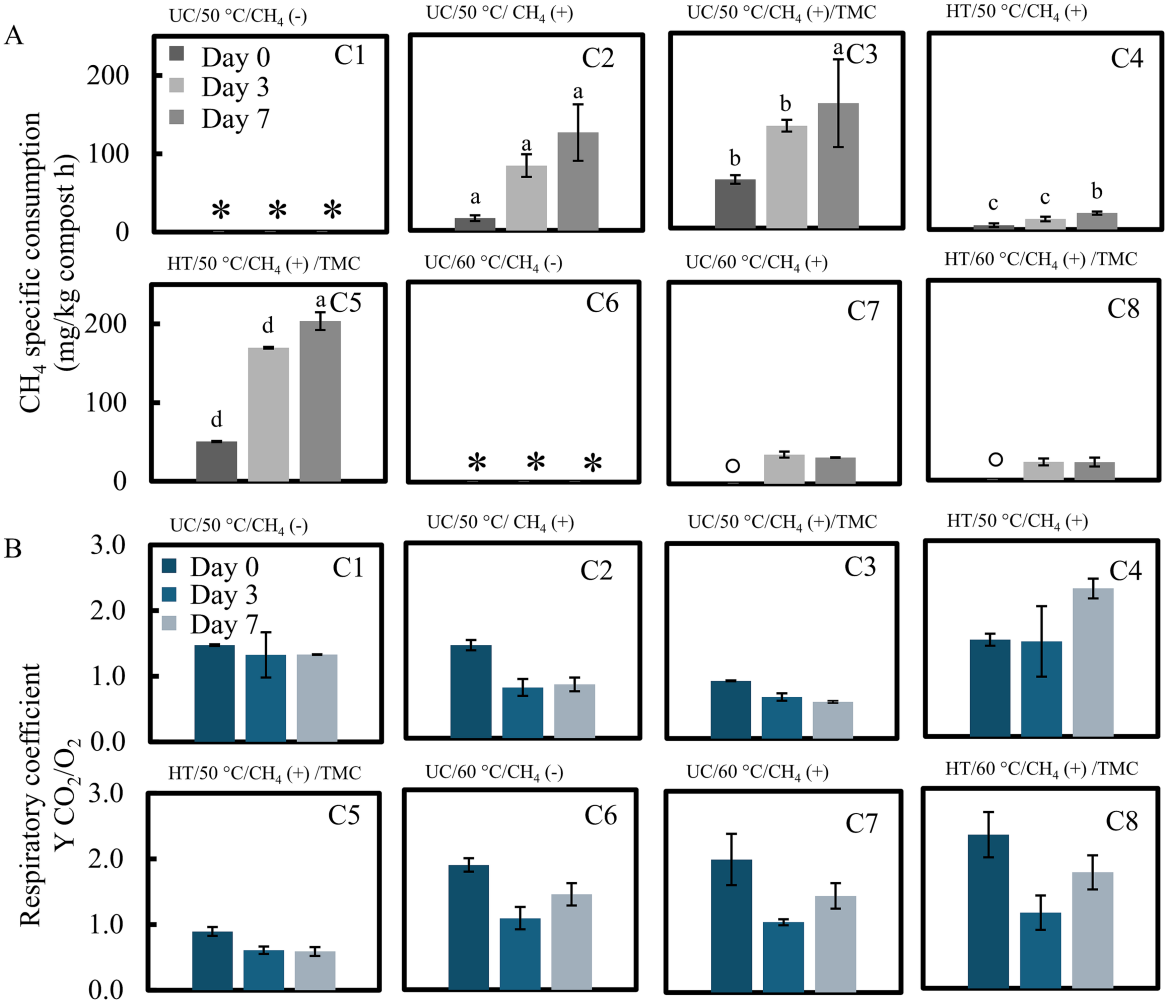
Characterization of methanotrophic activity in compost. **(A)** CH_4_ maximum rates, * no CH_4_ concentration, ° no CH_4_ consumption. Different letters indicate significant differences among treatments C2–C5 for each day according to LSD test (*p* < 0.05). **(B)** Respiratory coefficient (mol CO_2_ produced/mol O_2_ consumed). UC, Untreated compost; HT, heat treated compost; CH_4_ (+), added CH_4_; CH_4_ (−), no added CH_4_; TMC, compost inoculated with TMC.

The properties of the pruning-waste compost used are summarized in Supplementary Table S1. Soluble organic carbon and nitrogen determined from the original compost, yielded a C/N ratio of 4.2 ± 0.9 mg C/mg N, which may support a significant growth of microbial population (Wilshusen et al., 2004). Methanotrophic and respiratory activities of the compost under different conditions, as described in the Materials and Methods section, and intended for biofilter application, are presented in Figure 4. Compost processing typically reaches internal temperatures of 55–65 °C and, therefore, thermophilic heterotrophic microbial activity, including methanotrophy, is expected (Jäckel et al., 2005). Previous studies by Gómez-Borraz et al. (2025) have shown that compost-based CH_4_ biofiltration systems can reach temperatures above 50 °C due to heat accumulation from the highly exothermic CH_4_ oxidation.

Based on these observations, the compost exhibited both heterotrophic and methanotrophic activities at high temperatures. As shown in Figure 4A and Table 4, compared with the non-inoculated condition, initial startup activities differed markedly with rates of 15 ± 3 and 62 ± 5 mg CH_4_/kg compost·h for C2 and C3, respectively. However, after 7 days, CH_4_ oxidation rates in compost (C2) did not differ significantly from those observed in inoculated compost (C3), despite higher initial activity. Methane oxidation in the compost (C2) reached a rate of 124 ± 36 mg CH_4_/kg compost·h, while the compost inoculation (C3) resulted a rate of 160 ± 56 mg CH_4_/kg compost·h. This effect may be attributed to microbial competition, leading to reduced methanotrophic performance. Heat treatment (C4) reduced native methanotrophic activity to 20 ± 2 mg CH_4_/kg compost·h. Inoculation following heat treatment (C5) yielded the highest CH_4_ oxidation rate, reaching 203 ± 11 mg CH_4_/kg compost·h, after 7 days. Successive heat treatments (C4) markedly reduced native microbial activity, thereby minimizing potential competition and facilitating rapid colonization by the TMC. This resulted in higher CH_4_ oxidation rates with improved adaptation toward methanotrophic activity.

**Table 4:** CH_4_ maximum consumption rate in compost kinetics.

Experiment condition	Day 0	Day 3	Day 7
C1: UC, 50 °C, CH_4_ (−)	nd	nd	nd
C2: UC, 50 °C, CH_4_ (+)	15.5 ± 3.6	82.7 ± 14.5	124.9 ± 36.1
C3: UC, 50 °C, CH_4_ (−), TMC	62.3 ± 5.4	131.0 ± 7.5	159.9 ± 56.1
C4: HT, 50 °C, CH_4_ (+)	5.3 ± 2.2	13.3 ± 2.9	20.8 ± 1.9
C5: HT, 50 °C, CH_4_ (+), TMC	50.5 ± 0.7	169.6 ± 1.1	203.3 ± 11.2
C6: UC, 60 °C, CH_4_ (−)	nd	nd	nd
C7: UC, 60 °C, CH_4_ (+)	0	36.2 ± 3.8	32.5 ± 0.2
C8: HT, 60 °C, CH_4_ (+), TMC	0	22.8 ± 4.2	22.4 ± 5.4

UC, Untreated compost; HT, Heat treated compost; CH_4_ (+), added CH_4_; CH_4_ (−) no added CH_4_; TMC, compost inoculated. nd, not detected.

Respiratory coefficients, reflecting the relative contributions of methanotrophic and heterotrophic oxidation processes, are shown in Figure 4B. The oxidation of organic matter derived from cellulose and other polymers in compost, is represented by carbohydrate oxidation as: ${\mathrm{CH}}_{2}O+O_{2}\to {\mathrm{CO}}_{2}+H_{2}O$ , yields a theoretical CO_2_/O_2_ = 1. However, CH_4_ oxidation represented as ${\mathrm{CH}}_{4}+2\;O_{2}\to {\mathrm{CO}}_{2}+2\;H_{2}O$ results in CO_2_/O_2_ = 0.5. Conditions involving inoculation (C3 and C5) displayed rapid adaptation and stable respiratory coefficient related to methanotrophy (~0.6). In contrast, treatments lacking both inoculation and heat treatment exhibited higher CO_2_/O_2_ yields, indicating increased competition for O_2_ consumption by heterotrophic microbes. Moreover, nutrient availability, particularly nitrogen, plays an important role in sustaining methanotrophic activity (Bodelier and Laanbroek, 2004). Consequently, competition for both nutrients and O_2_may further contribute to reduced CH_4_ oxidation rates and instability of microbial populations. Additionally, methanotrophic activity was also detected at 60 °C (C6–C8), although rates showed a steep reduction than those observed at 50 °C. Elevated respiratory coefficients observed at 60 °C (C6–C8) were not consistent with the microbial activity measured under these conditions and may instead reflect CO_2|_ desorption at higher temperatures.

### 3.3 Biofiltration of CH_4_ at thermophilic conditions

**Figure 5:**
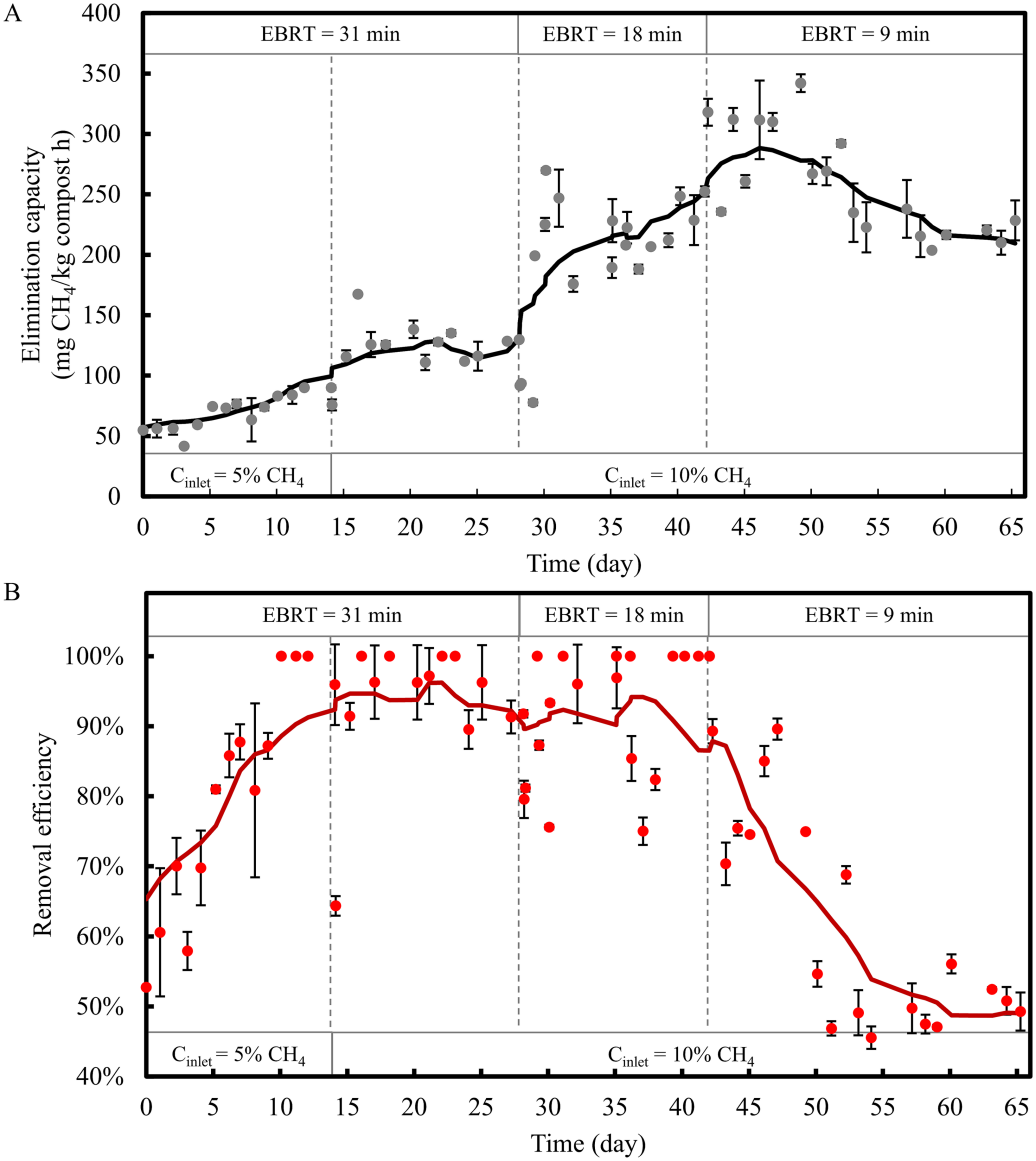
CH_4_ biofilter **(A)** elimination capacity and **(B)** removal efficiency during time course varying the CH_4_ input load.

The performance of the CH_4_ biofilter packed with heat treated compost and inoculated with the TMC is shown in Figure 5 (also see Supplementary Figure S5). Packing acclimatization enabled a rapid start-up, with an initial RE of approximately 50% that increased to 100% by day 10 at an inlet CH_4_ concentration of 5% and an EBRT of 31 min. A similar adaptation was observed when the inlet concentration was increased to 10% and the EBRT was reduced to 18 min, with RE increasing from approximately 65% on day 14 to 100% by day 16. Finally, the maximum EC of 342 ± 7.4 mg CH_4_/kg compost·h, corresponding to an RE of 75%, was achieved on day 49 with an EBRT of 9 min. Complete CH_4_ removal was achieved at IL of ~250 mg CH_4_/kg compost·h when operating at 10% CH_4_ and an EBRT of 18 min. As shown in Supplementary Figure S6, the EC–IL correlation indicated a maximum EC of approximately 253 mg CH_4_/kg compost·h with a constant RE close to 92%. A critical IL of 275 mg CH_4_/kg·h (equivalent to 198 g CH_4_/m^3^·h, assuming *ρ* = 0.72 kg/L) was identified, beyond which RE declined due to EC limitation.

**Figure 6:**
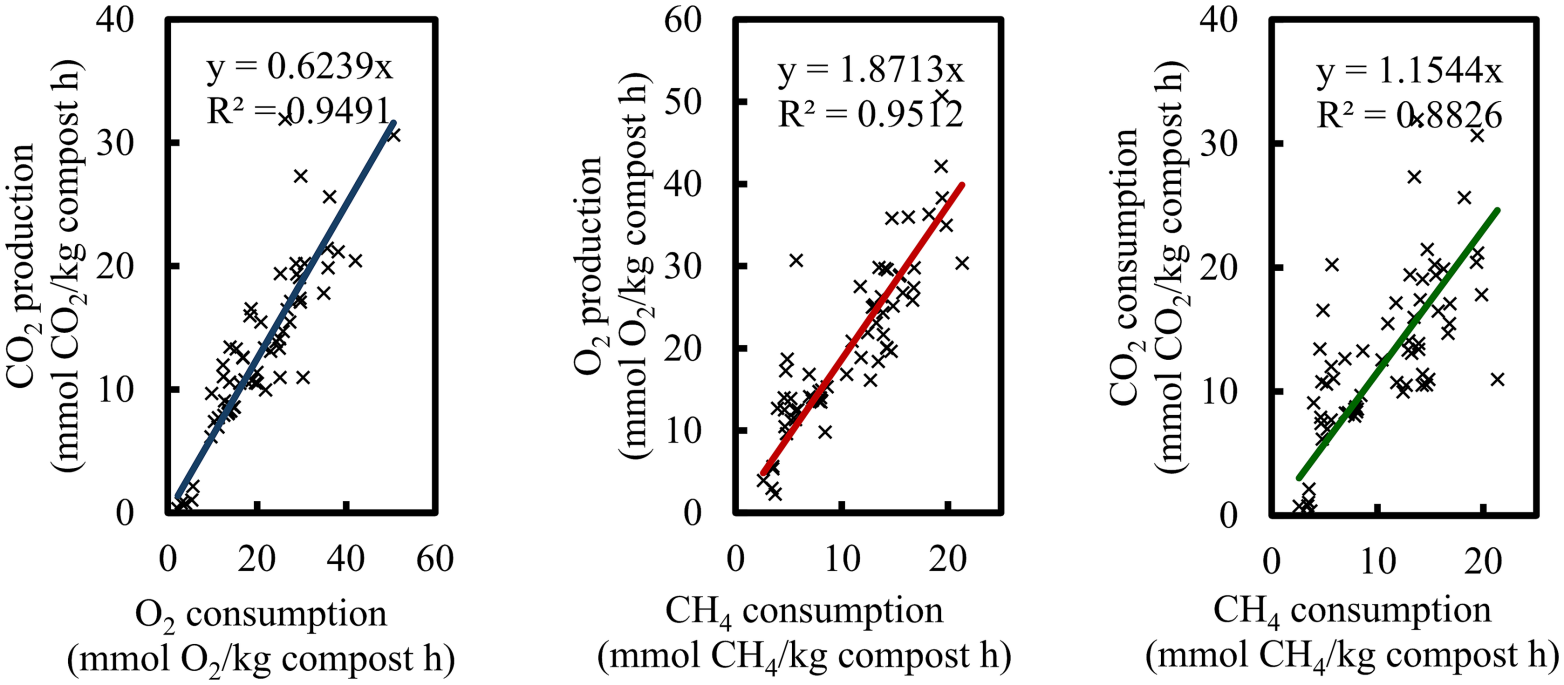
Correlation of O_2_ and CH_4_ consumption and CO_2_ production for coefficient respiratory (Y CO_2_/O_2_), CO_2_ yield (Y CO_2_/CH_4_), and O_2_ yield (Y O_2_/CH_4_).

Biofilter yields per CH_4_ consumed were 1.15 and 1.87 for CO_2_ and O_2_, respectively, resulting in a respiratory coefficient of 0.62, indicative of predominantly methanotrophic activity (Figure 6). Periods of reduced RE between days 35–40 coincided with outlet O_2_ concentrations below 5% (Supplementary Figure S7D). A subsequent decline in performance was observed after day 54, with EC decreasing to ~225 mg CH_4_/kg compost·h and RE to ~50%, which may be attributed to desiccation caused by increased temperature from high activity. During biofilter operation, the initial compost moisture content (~70%) decreased due to evaporation of water and was maintained at approximately 40% through periodic irrigation (Supplementary Figure S7A). Further, at the end of the biofilter operation, the moisture content of the compost remained at 40%, while the pH was 8.3, close to the initial pH of 8.15. Under thermophilic conditions, performance of biofilter was strongly influenced by the adequate control of operational parameters. Ultimately, performance was constrained by gas phase stoichiometry, as higher CH_4_ concentrations required increased O_2_ availability beyond that provided by air to satisfy the molar ratio for complete CH_4_ oxidation. Moreover, yield of CO_2_ greater than 1 indicate that heterotrophic metabolism contributed to a portion of both CO_2_ production and O_2_ consumption, thereby competing with methanotrophs for O_2_.

Prior heat treatment and subsequent inoculation enhanced CH_4_ removal in the biofilter by enabling rapid adaptation to high inlet loads. Different EBRTs and CH_4_ concentrations were evaluated to characterize the maximum EC. Reported EC values for mesophilic CH_4_ biofilters typically ranged between 20 and 70 g CH_4_/m^3^·h (Wilshusen et al., 2004; Nikiema et al., 2005; Kim et al., 2014), whereas thermophilic systems had achieved values between 40 and 100 g CH_4_/m^3^·h (Huete et al., 2018; Fjelsted et al., 2020; Gómez-Borraz et al., 2025). In the present study, a maximum EC of 246 g CH_4_/m^3^·h was achieved for 75% RE, while CH_4_ complete removal was maintained at 178 g CH_4_/m^3^·h (calculated using *ρ* = 0.72 kg compost/L). These results place the present system among the highest-performing thermophilic biofilters, even under conditions of complete CH_4_ oxidation. Microbial adaptation under thermophilic conditions improved performance, allowing both high EC and RE values. Nonetheless, the decline in performance observed after day 54, potentially attributable to processes such as desiccation, biomass accumulation, and channeling (Wilshusen et al., 2004; Revah and Morgan-Sagastume, 2005), highlights the need for careful control of operational parameters, particularly moisture content. Despite this decline, pH and moisture conditions remained within ranges previously reported as favorable for thermophilic CH_4_ biofilters (Fjelsted et al., 2020; Yang et al., 2021; Gómez-Borraz et al., 2025).

### 3.4 Effects of operational parameters on performance of thermophilic biofiltration

**Figure 7:**
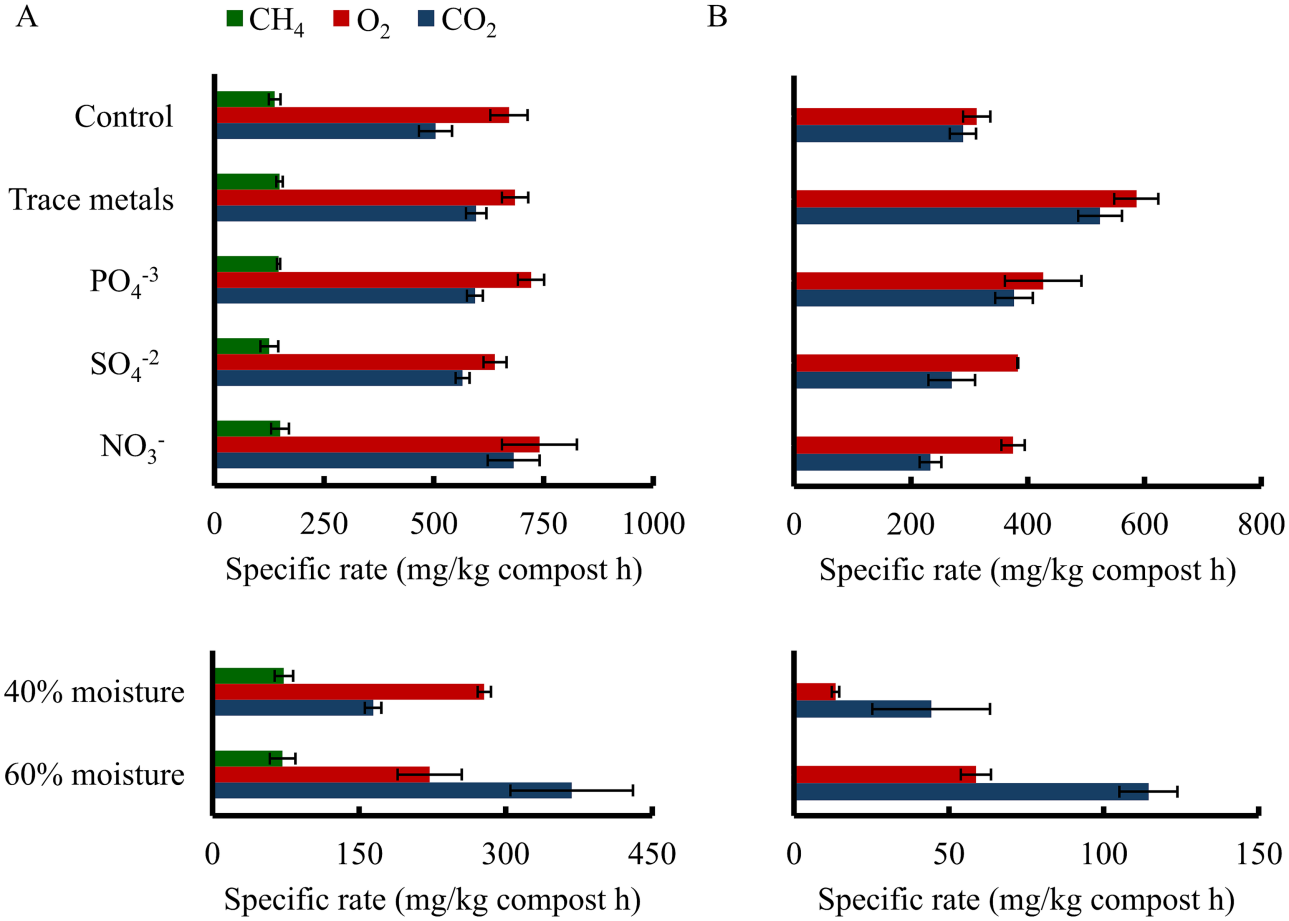
Effect of operational parameters on **(A)** methanotrophic and **(B)** heterotrophic activity on the biofilter compost used during 65 days of operation for CH_4_ removal.

The abiotic factors influencing thermophilic biofilter performance in active compost were further evaluated, as described in the Materials and Methods section, with results shown in Figure 7. Nutrient availability is another critical factor influencing microbial activity and consequent biofilter performance (Khabiri et al., 2020). In this study, nutrient supplementation did not enhance methanotrophic activity compared with the control (~140 mg CH_4_/kg compost·h), indicating that compost contained sufficient nutrients to support growth of microbes. The absence of clear nutrient limitations in the present study does not contradict previous reports (Nikiema et al., 2010; Veraart et al., 2015), as such limitations may arise from nutrient distribution within packing material rather than from bulk nutrient availability. Compost mixing has been shown to improve nutrient distribution and reduce localized nutrient limitations (Auria et al., 2000; Wilshusen et al., 2004). In contrast, supplementation with trace metals significantly stimulated heterotrophic activity without improving CH_4_ removal, suggesting that these elements primarily enhanced non-methanotrophic functions, such as biofilm formation, consistent with observations from the TMC bioreactor culture.

Compost moisture contents of 40 and 60% resulted in comparable CH_4_ oxidation rates under methanotrophic conditions, whereas heterotrophic activity was elevated, as evidenced by higher CO_2_ production and O_2_ consumption. Further, supplementation of TMC culture with additional carbon substrates (data not shown) did not alter the methanotrophic activity. Although most aerobic methanotrophs are unable to use carbohydrates as growth substrates, such amendments may influence the methanotrophic community by stimulating interactions with other microbial populations (Kim et al., 2014; Avila-Nuñez et al., 2024). Consequently, effective CH_4_ removal on organic packing material may depend not only on methanotroph populations but also on the functional interactions within the broader community (Ho et al., 2014). Overall, the observed effects of moisture and nutrients on microbial activity suggest that moisture and certain elements (trace metals) are the most relevant abiotic factors influencing heterotrophic microorganisms under the studied conditions.

## 4 Conclusion

This study provides evidence that thermophilic methane biofiltration can be effective (high EC), efficient (high RE), and robust (long term operation) when both microbial adaptation and key operational factors are carefully controlled. The results showed that preconditioning compost through heat treatment and selective inoculation with a thermophilic methanotrophic consortium accelerated the system start-up allowing sustained CH_4_ removal even under elevated inlet loads. These findings highlight that the successful deployment of thermophilic CH_4_ biofiltration requires the operational control of temperature, inlet load, moisture content, and nutrient availability.

Overall, this study contributes to present understanding and supports the feasibility of thermophilic biofiltration as an effective strategy for treating diffuse methane emissions in environments where elevated temperatures naturally occur, such as composting facilities, thermophilic anaerobic digestion, and landfill covers. Future work should focus on strategies to manage microbial community dynamics and overcome mass-transfer limitations, thereby enabling the development of more robust, scalable, and climate-relevant biofiltration systems.

## Data Availability

The original contributions presented in the study are included in the article and the supplementary material, further inquiries can be directed to the corresponding author/s.
