# Supplementary material for: Efficient methane removal by thermophilic methanotrophs in a compost biofilter
Source: Front Microbiol. 2026 Mar 25;17:1777601. doi: 10.3389/fmicb.2026.1777601 (PMC13057324; doi:10.3389/fmicb.2026.1777601)
Supplement: Supplementary file 1 [file Supplementary_file_1.docx]

Supplementary Material

**
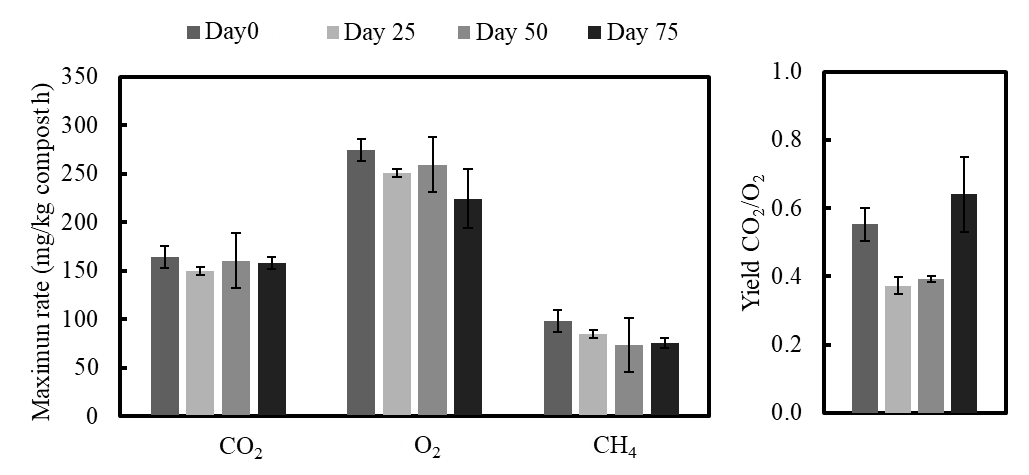
**

**Supplementary Figure 1.** Maximum consumption rate and respiratory coefficient during maintenance of compost inoculum with TMC to startup of biofilter.

**
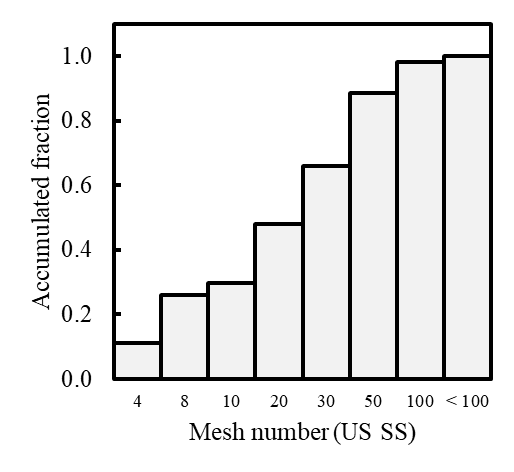
**

**Supplementary Figure 2.** Particle size accumulated distribution of compost source determined in mass fraction.


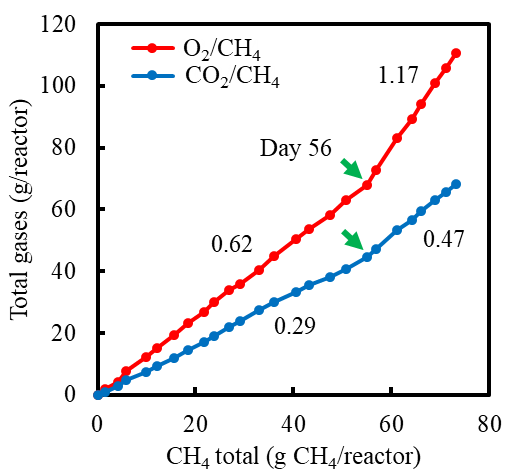


**Supplementary Figure 3.** Total gases – O_2_ consumption or CO_2_ production – against CH_4_ consumption in bioreactor operation with TMC. Arrow indicates the inflection point in yields related to metabolic changes from growth to stationary phase.

**
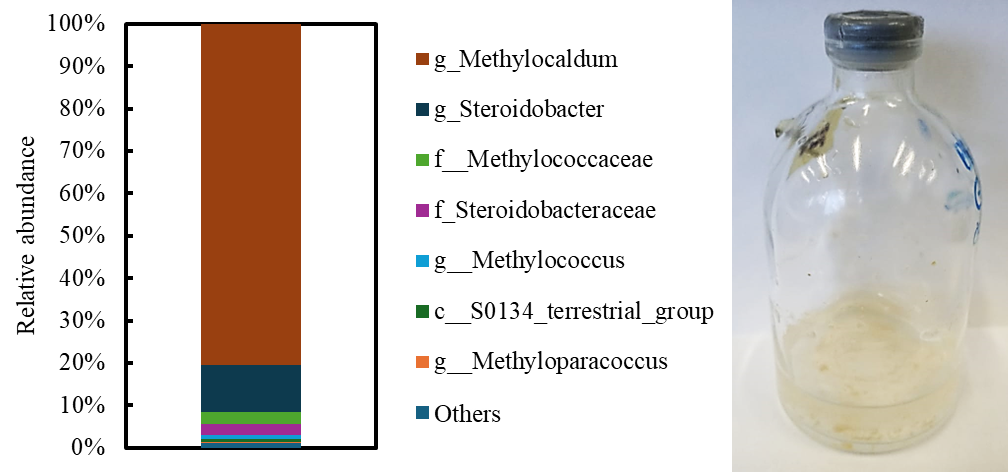
**

**Supplementary Figure 4.** Relative abundance of bacteria determined in TMC culture by 16S rRNA gene sequencing.


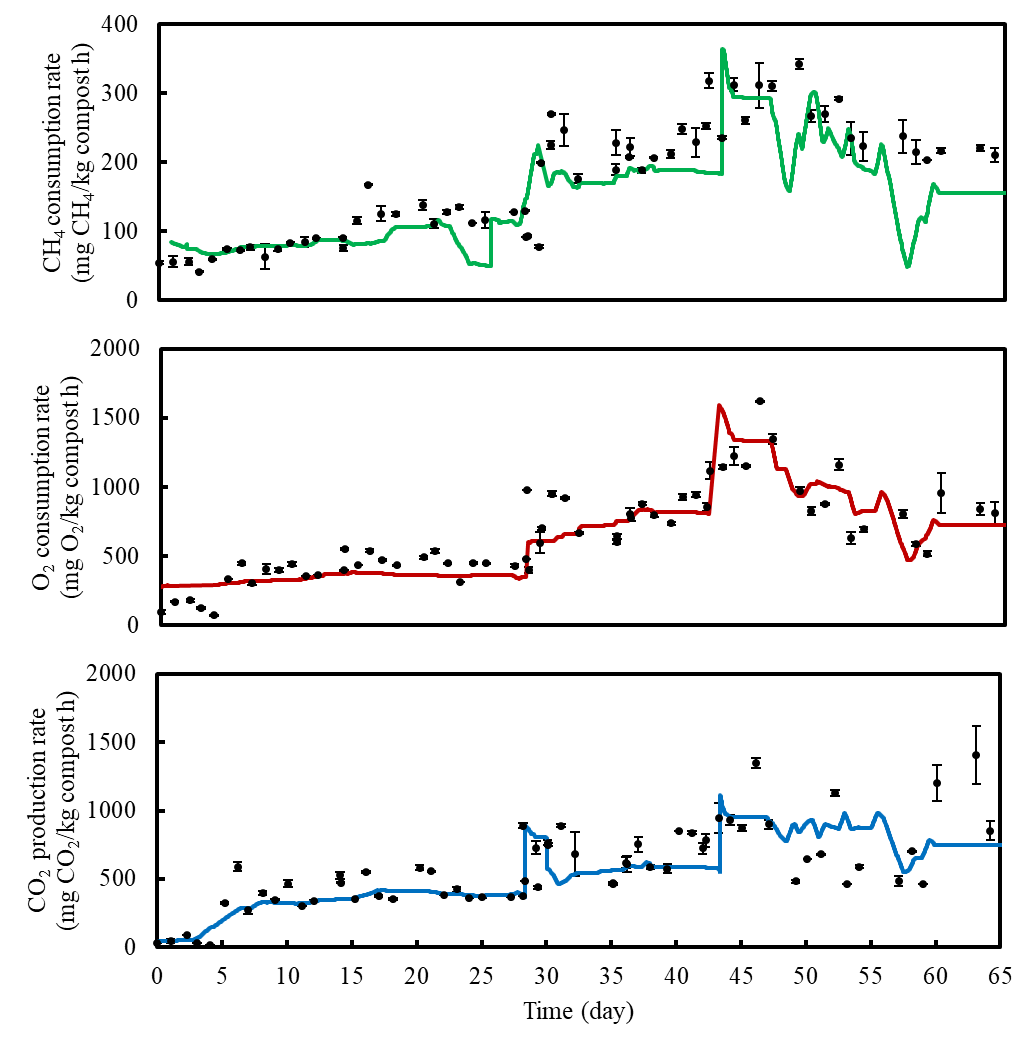


**Supplementary Figure 5.** CH_4_ and O_2_ consumption and CO_2_ production throughout the course of biofilter operation show the rate determined using the signal from the concentration sensors.

**
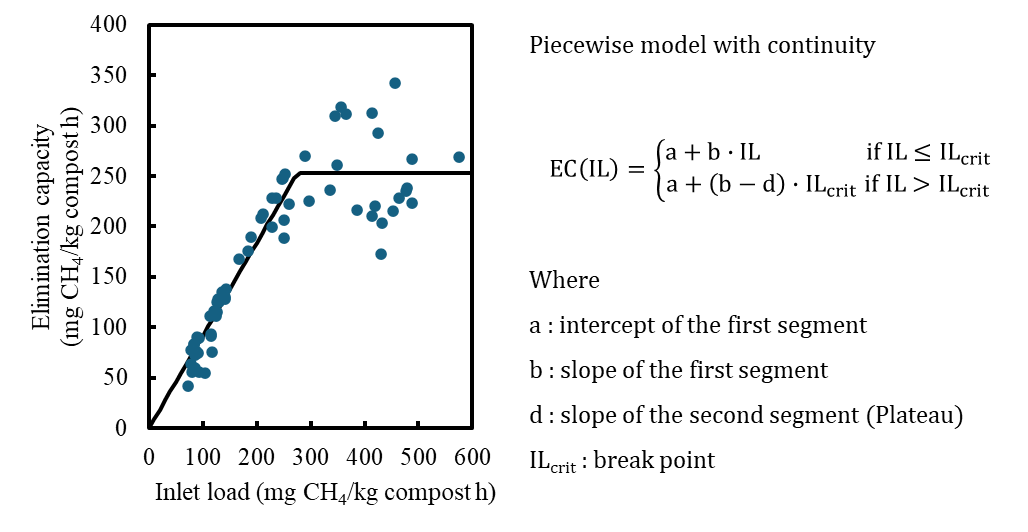
**

**Supplementary Figure 6.** EC and IL correlation fit to the piecewise model. Values of parameters are a = 0, b = 0.92, d = 0 and IL_crit_ = 275.82.


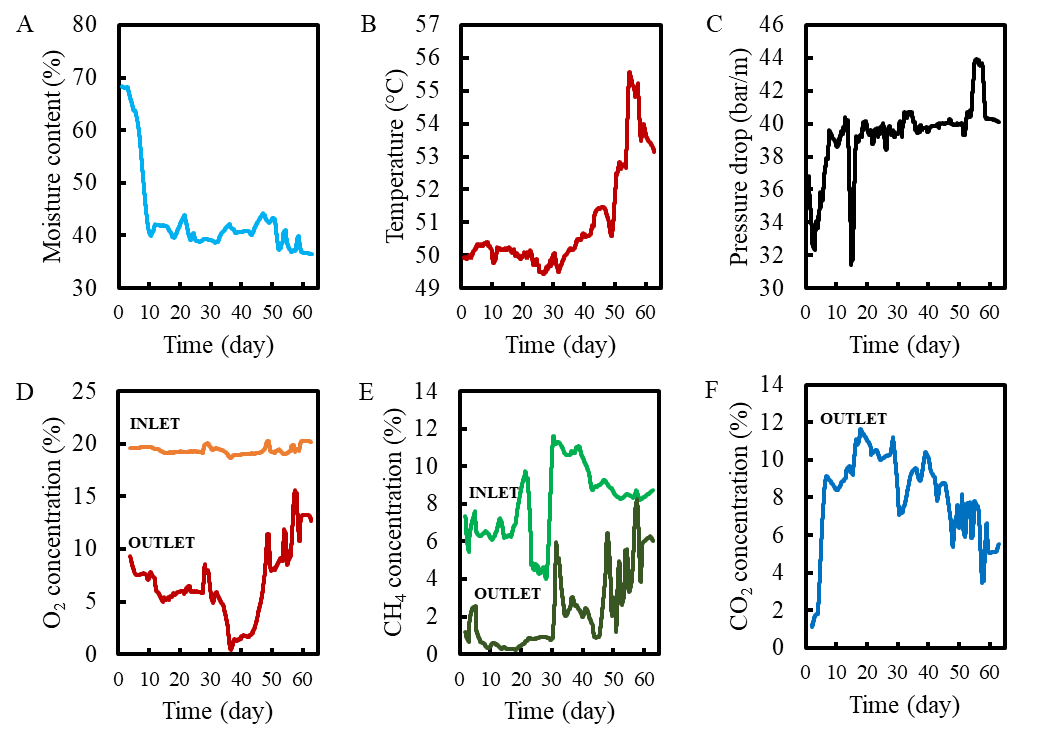


**Supplementary Figure 7.** Sensor signals throughout the course of biofilter operation for measurements: **(A)** moisture, **(B)** temperature, **(C)** pressure drop, **(D)** O_2_ concentration, **(E)** CH_4_ concentration and **(F)** CO_2_ concentration.

| **Supplementary Table 1.** Properties of compost from pruning waste utilized in experiments. | |
| --- | --- |
| Characteristic | Results |
| Moisture | 52 % |
| Apparent density | 0.48 kg/L |
| Packed density | 0.72 kg/L |
| Water holding capacity (WHC) | 2.1 L water/kg compost |
| Porosity* (ø) | 0.38 |
| pH | 8.15 |

*Porosity was calculated as $\emptyset=V_{total}-V_{solid}/V_{total}$.

| **Supplementary Table 2.** Technical specifications of sensors used in this study. | | | |
| --- | --- | --- | --- |
| Sensor | Model | Measurement range | Reference link |
| Electrochemical sensor O_2_ concentration | SEN0322 | 0 – 25% V | https://store.arduino.cc/products/gravity-i2c-oxygen-sensor |
| Electrochemical sensor CO_2_ concentration | MG-811 | 0 – 10% V* | https://store.arduino.cc/products/gravity-analog-co2-gas-sensor-mg-811-sensor |
| Analog sensor CH_4_ concentration | MQ4 | 0.2 – 10% V* | https://www.dfrobot.com/product-683.html |
| Digital barometric pressure sensor | MPS20N0040D-S | 0 – 40 KPa | https://uelectronics.com/producto/mps20n0040d-s-modulo-sensor-de-presion-barometrica |
| Digital temperature sensor | DS18B20 | - 55 – 125 °C | https://www.dfrobot.com/product-689.html |
| Analog capacitive soil moisture sensor | SEN-HS-CAP | 0 – 100% HR* | https://store.arduino.cc/products/gravity-analog-capacitive-soil-moisture-sensor-corrosion-resistant |

*Sensors calibrated under operating conditions for the indicated measurement range.
